# 疏肝肺积方结合心理干预对原发性肺癌患者生存质量的影响

**DOI:** 10.3779/j.issn.1009-3419.2012.01.06

**Published:** 2012-01-20

**Authors:** 逸临 姚

**Affiliations:** 200032 上海，上海中医药大学附属龙华医院肿瘤科 Department of Medical Oncology, Longhua Hospital Affiliated to Shanghai University of Traditional Chinese Medicine, Shanghai 200032, China

**Keywords:** 肺肿瘤, 疏肝肺积方, 心理干预, 生存质量, Lung neoplasms, Feiji decoction for soothing the liver, Psychotherapy, Quality of life

## Abstract

**背景与目的:**

原发性支气管肺癌是最常见的恶性肿瘤，带给患者巨大的躯体病痛和情绪障碍，而且明显降低患者的生存质量，本实验旨在研究疏肝肺积方结合心理干预对原发性肺癌患者生存质量、体能状态的影响。

**方法:**

将118例原发性非小细胞肺癌患者随机分为两组：疏肝肺积方结合心理干预加化疗组（联合治疗组57例）和单纯化疗组（61例）。联合治疗组采用疏肝肺积方和心理干预与化疗的联合应用；单纯化疗组仅接受化疗，共完成2次化疗，为1个总疗程。采用欧洲癌症研究与治疗组织生命质量核心量表（European Organization for Research and Treatment of Cancer QoL Questionnaire, EORTC QLQ-C30）和肺癌患者生命质量测定特异性模块（EORTC QLQ-LC13）测定生存质量，卡氏评分（Karnofsky performance status, KPS）和ECOG评分（East Cooperative Oncology Group performance status）测定体能状态。

**结果:**

联合治疗组生理、角色、情感、认知、社会功能以及总健康状况领域的评分比单纯化疗组的评分高，生存质量更好，且具有统计学意义（*P*＜0.01）；联合治疗组疲倦、恶心呕吐、疼痛、气促、失眠、食欲丧失、便秘症状以及肺癌特异症状评分比单纯化疗组的评分低，缓解更明显，且具有统计学意义（*P*＜0.01）；联合治疗组卡氏评分和ECOG评分提高和稳定的患者多于单纯化疗组，且具有统计学意义（*P*＜0.01）。

**结论:**

疏肝肺积方结合心理干预能缓解肺癌患者的临床症状、改善体能状态、提高生存质量，具有较好的临床疗效。

## Abstract (en)

**Background and objective:**

Primary lung cancer is one of the most common malignant tumors. It causes great pain and mood disorders to patients, and significantly reduces their quality of life. The aim of the current study is to evaluate the effect of Feiji Decoction for soothing the liver combined with psychotherapy on quality of life (QoL) and physical status of patients with primary lung cancer.

**Methods:**

A total of 118 patients with primary non-small cell lung cancer were randomly divided into two groups. The 57 patients in the combined therapy group were treated with Feiji Decoction for soothing the liver and psychotherapy combined with chemotherapy, whereas the 61 patients in the control group were treated with chemotherapy only. Both groups were observed for the two treatment courses. The European Organization for Research and Treatment of Cancer QoL Questionnaire LC-43 (EORTC QLQ-LC43) was used to assess the QoL of every patient in both groups before and after treatment scales. At the same time, physical status was assessed using the Karnofsky performance status (KPS) and East Cooperative Oncology Group performance status (ECOG).

**Results:**

The scores of physiology function, role function, emotion function, cognize function, society function, and general health in the therapy group were higher than that of the control group. The therapy group also showed better QoL results than the contol group. Significant differences were observed between the two groups (*P* < 0.01). Meanwhile, the scores of fatigue, vomit, pain, polypnea, insomnia, anorexia, constipation, and specific manifestation of lung cancer in the therapy group were obviously lower than that of the control group; more patients were observed to be relieved. Significant differences between the two groups were observed (*P* < 0.01). The KPS and ECOG scores of the patients were observed to have improved and stabilized in the therapy group than that of the control group; the differences were statistically significant (*P* < 0.01).

**Conclusion:**

Feiji Decoction for soothing the liver combined with psychotherapy can alleviate the clinical symptoms, elevate the physical status, and improve the QOL of patients with primary lung cancer. Thus, this therapy has a good clinical therapeutic effect.

肺癌是我国最常见的恶性肿瘤之一，但肺癌的治疗至今仍无突破性进展，除早期手术切除外，大部分中晚期患者中位生存期难以超过10个月，5年生存率低于10%，放疗、化疗的疗效并不理想且副作用较大，对于此类患者，提高生存质量、延长生存期应成为治疗的主要目的^[1]^。所以在临床综合治疗中，正逐渐将生存质量（quality of life, QoL）纳入并作为一个医疗结局评价点，因为它可以更多地反映患者的主观感受，帮助医生了解患者的生存状态和心理状态。WHO对生存质量的定义为：不同文化和价值体系中的个体对他们的目标、期望、标准以及所关心事情有关的生活状况的体验。本研究采用欧洲癌症研究与治疗组织生命质量核心量表（European Organization for Research and Treatment of Cancer QoL Questionnaire, EORTC QLQ-C30）和肺癌患者生命质量测定特异性模块（EORTC QLQ-LC13）测定生存质量，卡氏评分（Karnofsky performance status, KPS）和ECOG评分（East Cooperative Oncology Group performance status）测定体能状态，对疏肝肺积方结合心理干预治疗原发性支气管肺癌患者的临床疗效进行观察。

## 1 资料与方法

### 1.1 病例选择标准

#### 1.1.1 纳入标准

① 符合原发性支气管肺癌诊断标准^[2]^和原发性支气管肺癌分期标准^[3]^；②经临床、影像学、细胞学和病理学检查确诊的肺癌患者；③年龄为18岁-75岁；④准备进行化疗的肺癌患者；⑤预计生存期≥6个月；⑥KPS≥60分；⑦无严重肝肾功能损害、无智力障碍；⑧小学及小学以上文化程度；⑨愿意加入研究。

#### 1.1.2 排除标准

① 既往和目前有精神疾病和精神疾病家族史；②有智力或认知功能障碍患者；③有精神疾病药物或酒精依赖史；④伴有严重的并发症；⑤伴有其它系统严重疾病、需要专科治疗者；⑥依从性差。

#### 1.1.3 剔除标准

① 治疗中出现严重并发症或病情急剧恶化需采用紧急处理措施；②由于病情需要暂停化疗、连续化疗未完成2个周期；③由于各种原因未能完成疏肝肺积方结合心理干预及资料填写不全。

### 1.2 一般资料

本研究共入组140例原发性非小细胞肺癌患者，全部病例来源于上海中医药大学附属龙华医院及上海交通大学附属胸科医院。依据随机数字表将患者随机分为疏肝肺积方结合心理干预加化疗组（联合治疗组57例）和单纯化疗组（61例）。最终完成两次调查的病例为118例，22例由于各种原因退出研究，完成率84.3%。对两组患者治疗前的一般情况包括性别、年龄、婚姻状况、文化程度、职业、治疗费用、临床分期、病理类型进行统计学均衡性检验，无明显差异（*P*＞0.05）。

### 1.3 治疗方法

联合治疗组患者同时接受疏肝肺积方结合心理干预与化疗，单纯化疗组患者仅接受化疗，两组患者都给予必要的对症治疗，疼痛者按照WHO三阶梯原则给药；骨髓抑制达Ⅱ度及以上者，予重组人粒集落细胞刺激因子（granulocyte colony-stimulating factor, G-CSF）至恢复正常，化疗前予以必要的止吐药物。

#### 1.3.1 疏肝肺积方结合心理干预治疗

自患者入组化疗前3天，开始服用中药并进行心理干预，直至第2次化疗的第28天，为1个总疗程。

##### 1.3.1.1 中药治疗

以疏肝肺积方为基本方（生黄芪30 g，生白术15 g，北沙参15 g，石上柏30 g，七叶一枝花24 g，冰球子30 g，山萸肉12 g，仙灵脾15 g，玫瑰花9 g，八月札15 g，绿萼梅9 g），可酌情加减。

##### 1.3.1.2 心理干预

① 心理诱导教育：肿瘤相关知识，常见治疗（手术、放疗、化疗）的不良反应，有效病例的介绍；②行为训练：每日练习太极拳一次，约15 min；或进行放松训练、引导性想象一次，约15 min；③个体化心理治疗：因人而异施治，针对患者出现的不良心理表现如焦虑、抑郁等进行个别交谈，帮助患者认识问题，改善焦虑或抑郁情绪；④家庭成员配合治疗：向治疗组家属说明综合心理干预措施的意义，组织患者与家属共同面对疾病，分担不良情绪，并改善患者及家属的心理状态。

#### 1.3.2 化疗

采用NP方案：长春瑞滨25 mg/m^2^, d1, d8；顺铂75 mg/m^2^；或者GP方案：吉西他滨1, 000 mg/m^2^, d1, d8, d15；顺铂75 mg/m^2^；每28天为1个周期，2个化疗周期为1个总疗程。

### 1.4 观察指标及疗效评定

#### 1.4.1 生存质量

采用EORTC QLQ-C30和EORTC QLQ-LC13量表。入组化疗前3天测定1次，第2次化疗的第28天测定1次。功能子量表和总健康状况，得分越高代表生存质量越好；症状子量表，得分越低代表生存质量越好；肺癌特异性模块，得分越低代表生存质量越好。

#### 1.4.2 体能状态

采用卡氏评分和ECOG评分，按照分级标准评定患者治疗前后的状态，与生存质量量表同时填写。KPS评分治疗后较治疗前增加≥10者为提高，减少≥10者为下降，变化＜10者为稳定。ECOG评分治疗后较治疗前减少≥1分为改善，增加≥1分为下降，变化＜1分为稳定。

### 1.5 统计学方法

采用SPSS 13.0统计软件包建立数据库，并进行统计学处理。计量资料：配对、两组及多组资料的比较，经正态性检验和方差齐性检验后，如果服从正态分布和方差齐，采用t检验或F检验；如果不服从正态分布或方差不齐，则采用秩合检验。计数资料：满足卡方检验条件的，采用*χ*^2^检验；如果1＜*T*＜5的格子数超过总格子数的1/5时，则采用*Fisher's*精确概率检验。*P*＜0.05为差异有统计学意义。

## 2 结果

### 2.1 欧洲癌症研究与治疗组织生存质量量表EORTC QLQ-C43

#### 2.1.1 联合治疗组治疗前后EORTC QLQ-C43评分对照（表 1）

**1:** 联合治疗组治疗前后EORTC QLQ-C43评分比较 The comparison of EORTC QLQ-C43 score before and after treatment of combination-therapy group

Items	Before treatment	After treatment	*t*	*P*
PF	61.29±18.96	72.51±14.59	5.97	< 0.001
RF	54.97±30.53	70.18±24.55	4.38	< 0.001
EF	67.54±23.24	89.62±10.83	6.90	< 0.001
CF	71.64±23.14	88.60±12.66	6.03	< 0.001
SF	48.54±24.05	58.48±22.96	6.03	< 0.001
GH	44.59±19.64	61.11±16.91	6.18	< 0.001
Fatigured	53.22±25.09	32.94±17.05	6.41	< 0.001
Vomit	25.44±25.02	6.14±12.05	5.70	< 0.001
Pain	28.95±22.39	11.99±16.29	5.32	< 0.001
Polypnea	35.09±21.29	28.65±20.35	2.03	0.047
Insomnia	38.01±23.94	16.96±20.04	7.40	< 0.001
Anorexia	34.50±29.52	9.36±15.11	6.86	< 0.001
Constipation	20.47±20.66	11.11±17.06	3.42	0.001
Diarrhea	8.19±14.48	2.92±9.51	2.88	0.006
Economic	49.71±27.55	45.03±27.09	1.27	0.209
QLQ-LC13	23.66±9.02	15.65±8.07	7.917	< 0.001
PF: physiological function; RF: role function; EF: emotion function; CF: cognitive function; SF: society function; GH: general health.				

可见联合治疗组患者经治疗后，在功能子量表中，角色、情感、认知以及总健康状况领域的得分升高较其它领域明显，生存质量更好。在症状子量表中，疲倦、恶心呕吐、失眠以及食欲丧失的得分下降较其它领域明显，生存质量更好。对各领域的评分进行配对t检验后可见生理、角色、情感、认知、社会功能以及总健康状况治疗后较治疗前评分升高，疲倦、恶心呕吐、疼痛、失眠、食欲丧失、便秘、腹泻以及肺癌特异症状治疗后较治疗前评分下降，生存质量明显提高，有统计学意义（*P*＜0.01）。

#### 2.1.2 单纯化疗组治疗前后EORTC QLQ-C43评分对照（表 2）

**2:** 单纯化疗组治疗前后EORTC QLQ-C43评分比较 The comparison of EORTC QLQ-C43 score before and after treatment of chemotherapy group

Items	Before treatment	After treatment	*t*	*P*
PF	68.63±17.94	53.01±24.14	7.74	< 0.001
RF	60.66±25.65	45.08±34.34	3.30	0.002
EF	73.63±23.77	57.24±26.85	5.38	< 0.001
CF	77.05±18.30	63.66±24.44	5.08	< 0.001
SF	54.37±24.70	41.26±29.44	3.61	0.001
GH	49.73±20.58	35.79±19.45	5.89	< 0.001
Fatigured	46.08±23.56	62.84±28.25	-5.14	< 0.001
Vomit	13.93±17.26	46.99±28.79	-9.64	< 0.001
Pain	26.50±25.71	32.51±24.99	-1.94	0.057
Polypnea	32.24±21.91	43.72±25.49	-4.27	< 0.001
Insomnia	36.61±27.69	54.10±35.05	-3.99	< 0.001
Anorexia	29.51±26.60	64.48±32.13	-7.24	< 0.001
Constipation	19.13±21.48	37.16±31.68	-4.67	< 0.001
Diarrhea	7.65±14.13	9.84±19.57	-0.73	0.470
Economic	47.54±33.59	59.56±31.69	-2.97	0.004
QLQ-LC13	20.87±10.02	27.87±14.35	5.238	< 0.001

可见单纯化疗组患者经治疗后，在功能子量表中，生理功能、角色功能、情感功能、认知功能、社会功能以及总健康状况的评分都有不同程度的下降，生存质量更差。在症状子量表中，恶心呕吐、失眠以及食欲丧失的得分升高较其它领域明显，生存质量更差。对各领域的评分进行配对t检验后得到表 2，可见生理、角色、情感、认知、社会功能以及总健康状况较治疗前明显下降，疲倦、恶心呕吐、气促、失眠、食欲丧失、便秘、经济困难以及肺癌特异症状较治疗前明显加重，生存质量明显降低，有统计学意义（*P*＜0.01）。疼痛、腹泻症状治疗后较治疗前评分上升，但无明显差异（*P*＞0.05）。

#### 2.1.3 两组患者治疗后EORTC QLQ-C43组间比较（表 3）

**3:** 联合治疗组与单纯化疗组治疗后的EORTC QLQ-C43组间对比 The comparison of EORTC QLQ-C43 score after the treatment of combination-therapy group and chemotherapy group

Item	Group	Mean	SD	*t*	*P*
PF	Combination-therapy	72.51	14.59	4.415	< 0.001
	Chemotherapy	53.01	24.14		
RF	Combination-therapy	70.18	24.55	4.008	< 0.001
	Chemotherapy	45.08	34.34		
EF	Combination-therapy	89.62	10.83	6.984	< 0.001
	Chemotherapy	57.24	26.85		
CF	Combination-therapy	88.60	12.66	5.88	< 0.001
	Chemotherapy	63.66	24.44		
SF	Combination-therapy	58.48	22.96	3.236	0.001
	Chemotherapy	41.26	29.44		
GH	Combination-therapy	61.11	16.91	6.286	< 0.001
	Chemotherapy	35.79	19.45		
Fatigured	Combination-therapy	32.94	17.05	5.759	< 0.001
	Chemotherapy	62.84	28.25		
Vomit	Combination-therapy	6.14	12.05	7.949	< 0.001
	Chemotherapy	46.99	28.79		
Pain	Combination-therapy	11.99	16.29	4.739	< 0.001
	Chemotherapy	32.51	24.99		
Polypnea	Combination-therapy	28.65	20.35	3.444	0.001
	Chemotherapy	43.72	25.49		
Insomnia	Combination-therapy	16.96	20.04	5.792	< 0.001
	Chemotherapy	54.10	35.05		
Anorexia	Combination-therapy	9.36	15.11	8.172	< 0.001
	Chemotherapy	64.48	32.13		
Constipation	Combination-therapy	11.11	17.06	4.861	< 0.001
	Chemotherapy	37.16	31.68		
Diarrhea	Combination-therapy	2.92	9.51	2.164	0.03
	Chemotherapy	9.84	19.57		
Economic	Combination-therapy	45.03	27.09	2.487	0.013
	Chemotherapy	59.56	31.69		
QLQ-LC13	Combination-therapy	15.65	8.07	5.748	< 0.001
	Chemotherapy	27.87	14.35		

可见通过治疗，联合治疗组患者的生理、角色、情感、认知、社会功能以及总健康状况比单纯化疗组评分高，生存质量更高，且具有统计学意义（*P*＜0.01）；疲倦、恶心呕吐、疼痛、气促、失眠、食欲丧失、便秘症状以及肺癌特异症状评分比单纯化疗组评分低，缓解更明显，生存质量更高且具有统计学意义（*P*＜0.01）。

### 2.2 体能状态

#### 2.2.1 KPS评分

将两组治疗前后KPS差值进行比较见表 4，可见联合治疗组与单纯化疗组治疗前后KPS差值变化有统计学意义（*P*＜0.01），联合治疗组KPS评分提高和稳定的患者多于单纯化疗组KPS评分提高和稳定的患者，表明疏肝肺积方结合心理干预加化疗组经治疗后体能状态更好。

**4:** 联合治疗组与单纯化疗组治疗前后KPS差值比较 The comparison of KPS difference before and after the treatment of combination-therapy group and chemotherapy group

Group	Increase	Stable	Decrease	Total	*χ*^2^	*P*
Combination-therapy	30	24	3	57	63.044	< 0.001
Chemotherapy	1	17	43	61		
Total	31	41	46	118		

#### 2.2.2 ECOG评分

将两组治疗前后ECOG差值进行比较见表 5。可见联合治疗组与单纯化疗组的治疗前后ECOG差值变化有统计学意义（*P*＜0.01），联合治疗组ECOG评分改善和稳定的患者多于单纯化疗组ECOG评分改善和稳定的患者，表明疏肝肺积方结合心理干预加化疗组经治疗后体能状态更好。

**5:** 联合治疗组与单纯化疗组治疗前后ECOG差值比较 The comparison of ECOG difference before and after the treatment of combination-therapy group and chemotherapy group

Group	Increase	Stable	Decrease	Total	*χ*^2^	*P*
Combination-therapy	19	35	3	57	64.942	< 0.001
Chemotherapy	1	13	47	61		
Total	20	48	50	118		

## 3 讨论

原发性支气管肺癌是目前世界上最常见的恶性肿瘤，2/3的患者在确诊时已属中晚期，失去了手术机会，尤其是非小细胞肺癌的放疗和化疗敏感性差且副作用多，在治疗中给患者带来了巨大的躯体病痛和情绪障碍，这不仅可以引起患者的生存质量下降，而且足以导致其生物学改变。随着健康观和医学模式的转变，传统的仅仅关注生命的延长和局部躯体功能改善的一些方法和评价指标体系面临严重挑战：一则未能表达健康的全部内涵，二则未能体现具有生物、心理和社会属性的人的整体性和全面性^[4]^。生活质量是顺应医学模式从单纯生物医学模式向生物——心理——社会医学模式转变而产生的一类新的健康和疾病变化的评价指标，更能全面反映人体的健康状况。越来越多的研究者认为适当的心理社会干预对患者的恢复是有益的，它可以改善个体的应对能力，减少情绪上的烦恼和情感上的孤独，从而改善患者的心理功能，以及在一定程度上缓解症状，最终提高其生存质量，甚至延长个体的存活时间。

七情是人体对客观事物的不同反应，正常情况下不会导致疾病的发生，但当这种刺激作用过强，或骤然发生，或经久不息，超过了人体本身的正常生理活动范围，才会影响健康导致疾病的发生。《古今医通》云：“七情不舒，遂成郁结，既郁之久，变病多端”。突然、强烈或持久的不良情绪更是影响患者生存质量、加重疾病的重要因素，这在肿瘤的病因病机中体现尤为明显。早在《素问·通评虚实论篇》中对噎嗝（指现代医学的食管癌）的发病原因的认识是：“膈塞闭绝，上下不通，则暴忧之病也。”《妇人良方》中认为乳岩（指现代医学的乳腺癌）的发生“此属肝脾郁怒，气血亏损。”李延在《医学入门》中云：“瘤初起如梅李，皮嫩而光，渐入石榴瓜瓠之状，原因七情劳欲，复被外邪，生痰聚瘀，随气留住，故有日瘤獒，总皆气血凝滞结成。”由此可见，情志表现过度、七情内伤必然会导致脏腑功能失调，气机升降不畅，久则脏腑亏虚，气滞血瘀，痰凝毒结，癌瘤形成。辛海等^[5]^根据对恶性肿瘤并发的情志症各种症状的中医辨证分析，认为肝郁气滞证、肝郁脾虚证、肝郁痰阻证和心脾两虚证最常见。谢永宏^[6]^应用健脾益气、养心安神中药调理改善肺癌术后患者的精神心理状态和生活质量，提高免疫调节功能。张四方等^[7]^应用益气健脾清热解毒中药治疗肺癌化疗间歇期患者，能明显改善肺癌患者的负性情绪，提高生活质量。

基于以上理论，本研究将疏肝肺积方与心理干预措施相结合，运用于原发性支气管肺癌患者，对治疗后患者的生存质量量表及体能状态评分进行疗效评价。结果表明：疏肝肺积方结合心理干预法能明显改善患者的心理和社会功能，缓解肺癌患者的常见临床症状，提高生存质量，加速化疗后患者功能状态的恢复及症状的缓解，具有较好的临床疗效。在功能领域中，尤其以角色、认知和总健康状况的改善最为明显；在症状方面，以疲倦、恶心呕吐、失眠和食欲丧失的改善尤为明显，这些疗效的产生与疏肝肺积方和心理行为干预措施的同时运用密不可分。这与文献报道一致，林丽珠等^[8]^发现，中医药对于化疗的毒副反应有一定的拮抗作用，在一定程度上维护并提高了患者的生存质量。王俊等^[9]^对乳腺癌术后化疗患者采用心理社会干预措施，包括健康教育、放松训练及集体心理干预，结果表明干预组患者的不良情绪明显改善，生存质量明显提高。本研究在对体能状态的观察中发现：疏肝肺积方结合心理干预加化疗组在经过了2次化疗后，患者的KPS评分分布呈明显上升趋势、ECOG评分分布呈明显下降趋势，而单纯化疗组在经过了两次化疗后，患者的KPS评分分布呈明显下降趋势、ECOG评分分布呈明显上升趋势。且将两组患者治疗前后KPS和ECOG差值变化进行比较，均有明显差异（*P*＜0.01），表明疏肝肺积方结合心理干预治疗能明显改善患者化疗后的体能状态，并有助于患者体能状态的恢复。

由此可见，疏肝肺积方结合心理干预能缓解肺癌患者的临床症状、改善体能状态、提高生存质量，具有较好的临床疗效。在治疗过程中，患者能更清楚地了解治疗的过程、目的及意义，明确良好的情绪与健康的关系，消除恐惧心理和不良认知，减轻心理困惑，增加对疾病的可控制感，调动患者潜在的解决自身问题的能力，减轻化疗引起的心理生理反应，增加机体免疫功能，抑制肿瘤增长；同时，确立了良好的医患关系，使患者能积极配合治疗，达到最佳治疗效果，从而提高生存质量。
